# Supplementary material for: Mitochondrial arginase-2 is essential for IL-10 metabolic reprogramming of inflammatory macrophages
Source: Nat Commun. 2021 Mar 5;12:1460. doi: 10.1038/s41467-021-21617-2 (PMC7936006; doi:10.1038/s41467-021-21617-2)
Supplement: Supplementary file 1 — Supplementary Information [file 41467_2021_21617_MOESM1_ESM.pdf]

Supplementary Information for:

Mitochondrial arginase-2 is essential for IL-10 metabolic reprogramming of inflammatory macrophages

Jennifer K. Dowling[1,2,3,4,10], Remsha Afzal[1,10], Linden J. Gearing[2,4], Mariana P. Cervantes-Silva[1], Stephanie Annett[1], Gavin M. Davis[6], Chiara De Santi[1], Nadine Assmann[6,7], Katja Dettmer[8], Daniel J. Gough[4,5], Glenn R. Bantug[7,9], Fidinny I. Hamid[4,5], Frances K. Nally[1], Conor P. Duffy[1], Aoife L. Gorman[6], Alex M. Liddicoat[6], Ed C. Lavelle[6], Christoph Hess[7,9], Peter J. Oefner[8], David K. Finlay[6], Paul J. Hertzog[2,4], Annie M. Curtis[1], Gavin P. Davey[6], Tracy Robson[1], Bryan R.G. Williams[4,5], and Claire E. McCoy[1,3,4,5**]

1 School of Pharmacy and Biomolecular Sciences, Royal College of Surgeons in Ireland, Dublin 2, Ireland.

2 Centre for Innate Immunity and Infectious Diseases, Hudson Institute of Medical Research, Clayton, VIC, Australia

3 FutureNeuro, SFI Research Centre, Dublin 2, Ireland.

4 Department of Molecular and Translational Science, Monash University, Clayton, VIC, Australia.

5 Centre for Cancer Research, Hudson Institute of Medical Research, Clayton, VIC, Australia.

6 School of Biochemistry and Immunology, Trinity College Dublin, Dublin, Ireland.

7 Immunobiology Laboratory, Department of Biomedicine, University Hospital Basel, Basel, Switzerland.

8 Institute of Functional Genomics, University of Regensburg, Regensburg, Germany.

9 Cambridge Institute of Therapeutic Immunology & Infectious Disease, Department of Medicine, University of Cambridge, Cambridge, UK.

10 These authors contributed equally.

** Lead Contact and corresponding author

Contents:

Supplementary Figure 1

Supplementary Figure 2

Supplementary Figure 3

Supplementary Figure 4

Supplementary Figure 5

Supplementary Table 1 - List of used antibodies, bacterial strains and cell lines

Supplementary Table 2 - List of primers

Supplementary Table 3 - List of used chemical reagents

Supplementary Table 4 – List of used commercial kits

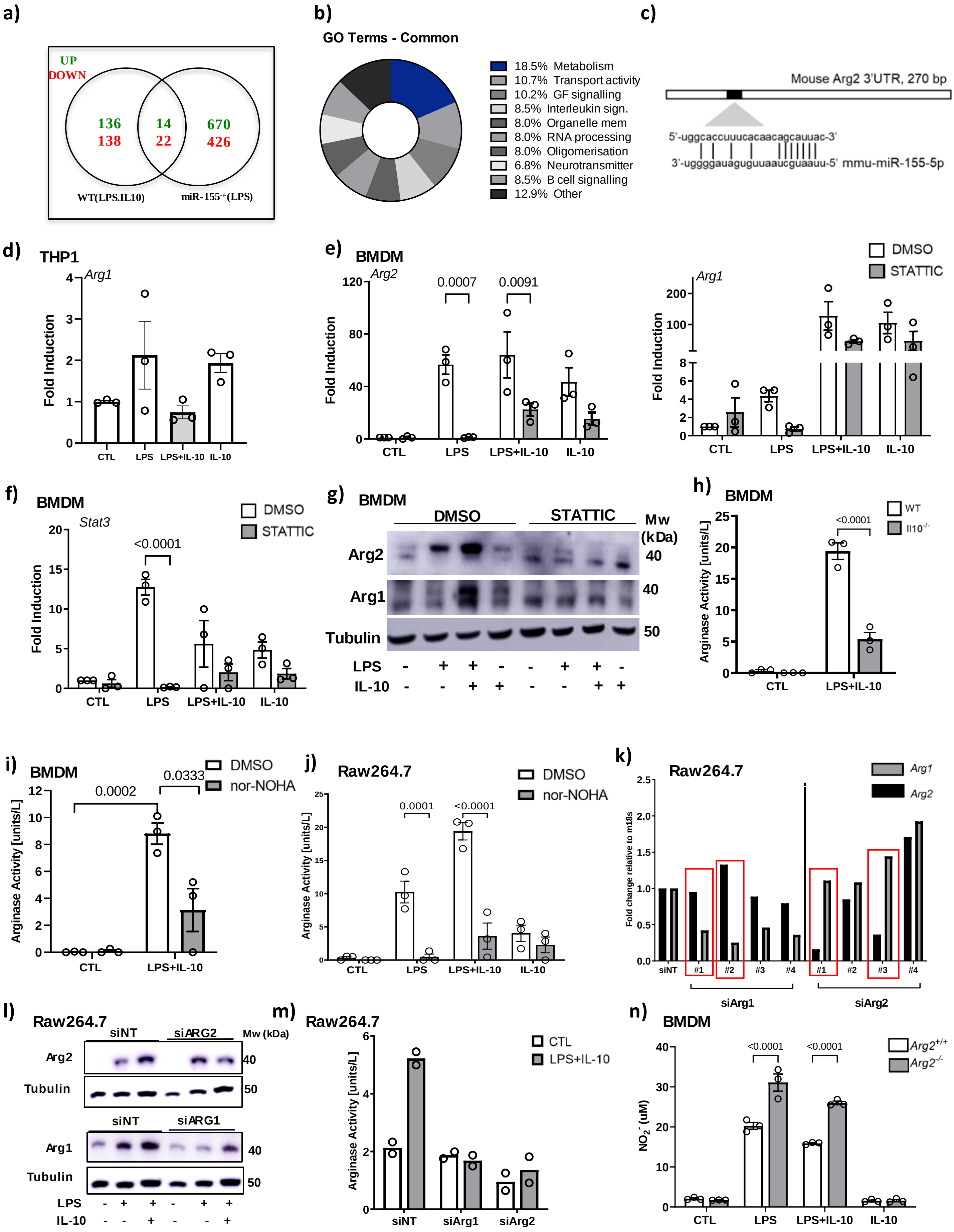
a)
UP
DOWN
136
138
14
22
670
426
WT(LPS.IL10)
miR-155-/-(LPS)
b)
GO Terms - Common
18.5% Metabolism
10.7% Transport activity
10.2% GF signalling
8.5% Interleukin sign.
8.0% Organelle mem
8.0% RNA processing
8.0% Oligomerisation
6.8% Neurotransmitter
8.5% B cell signalling
12.9% Other
c)
Mouse Arg2 3'UTR, 270 bp
5'-uggcaccuuucacaacagcauuac-3'
3'-uggggauaguguuaaucguaauu-5' mmu-miR-155-5p
d)
THP1
Arg1
Fold Induction
CTL
LPS
LPS+IL-10
IL-10
e)
BMDM
Arg2
Fold Induction
0.0007
0.0091
CTL
LPS
LPS+IL-10
IL-10
DMSO
STATTIC
Arg1
Fold Induction
CTL
LPS
LPS+IL-10
IL-10
f)
BMDM
Stat3
DMSO
STATTIC
Fold Induction
<0.0001
CTL
LPS
LPS+IL-10
IL-10
g)
BMDM
DMSO
STATTIC
Mw (kDa)
Arg2
Arg1
Tubulin
40
40
50
LPS - + + - - + + -
IL-10 - - + + - - + +
h)
BMDM
WT
Il10-/-
<0.0001
Arginase Activity [units/L]
CTL
LPS+IL-10
i)
BMDM
DMSO
nor-NOHA
0.0002
0.0333
Arginase Activity [units/L]
CTL
LPS+IL-10
j)
Raw264.7
DMSO
nor-NOHA
0.0001
<0.0001
Arginase Activity [units/L]
CTL
LPS
LPS+IL-10
IL-10
k)
Raw264.7
Arg1
Arg2
Fold change relative to m18s
siNT
#1
#2
#3
#4
#1
#2
#3
#4
siArg1
siArg2
l)
Raw264.7
siNT
siARG2
Mw (kDa)
Arg2
Tubulin
40
50
siNT
siARG1
Arg1
Tubulin
40
50
LPS - + + - + +
IL-10 - - + - - +
m)
Raw264.7
CTL
LPS+IL-10
Arginase Activity [units/L]
siNT
siArg1
siArg2
n)
BMDM
Arg2+/+
Arg2-/-
<0.0001
<0.0001
NO2- (uM)
CTL
LPS
LPS+IL-10
IL-10

Supplementary Figure 1. Arginase-2 is an IL-10/miR-155 regulated gene. (a) Venn diagram illustrating gene number changes when WT(LPS) genes were compared to WT(LPS+IL10) and *miR-155*$^{-/-}$(LPS) genes. **(b)** Common gene ontology (GO) terms identified using gene set enrichment analysis (ROMER) on WT(LPS+IL10) and *miR-155*$^{-/-}$ (LPS) anti-inflammatory genes. **(c)** The miR-155 binding site (black box) in the 3′-UTR of mouse *Arg-2* mRNA. The sequence of mouse miR-155 is shown aligned with its predicted target site in the 3′-UTR of *Arg2* mRNA. **(d-n)** Cells were left unstimulated, or stimulated with LPS, LPS+IL-10, or IL-10 alone for 24 hours (or for 8 hours after STATTIC pre-treatment in **(e-g)**): **(d)** RT-PCR analysis of *Arg1* mRNA levels in THP-1 cells. Data points represent n=3 independent experiments. **(e)** RT-PCR analysis of **(e)** *Arg2*, *Arg1*, (**f)** *Stat3* mRNA levels, and **(g)** Arg2, Arg1 protein levels in BMDM. **(e,f)** Data points represent average of technical triplicates over n=3 independent experiments. **(g)** Representative of two independent experiments. **(h-j)** Urea measured as output of arginase activity (n=3 biological independent experiments) in **(h)** WT (*Il10*$^{+/+}$) vs *Il10*$^{-/-}$ BMDM, **(i)** DMSO vs nor-NOHA treated BMDM, **(j)** DMSO vs nor-NOHA treated Raw 264.7 cells. **(k-m)** Raw 264.7 macrophages were transfected with different Accell® siRNA for 48 hours against *Arg1* and *Arg2* (siArg1, siArg2) and compared to non-targeting siRNA (siNT)). Samples were then stimulated with LPS+IL-10 for 24 hours, and looked at for: **(k)** *Arg1* and *Arg2* mRNA. siRNA shown in red boxes were used for later knockdown experiments (n=1 independent experiment to test for siRNA efficacy). **(l)** Protein expression of Arg1 and Arg2 in Raw 264.7. Representative of two independent experiments. **(m)** Urea as output of arginase activity. Data points show average of technical triplicates over n=2 independent experiments. **(n)** NO production in *Arg2*$^{+/+}$ vs *Arg2*$^{-/-}$ BMDM (n=3 biological experiments). **(d-f, k)** Data are normalized to *m18s*, and expressed as fold change relative to controls **(d-f, h-j, n)** Data shown is mean with error bars representing +/- SEM. Data is compared using two-way ANOVA with Sidak's multiple comparison post-hoc test. P-values are indicated on graphs wherever statistically significant.

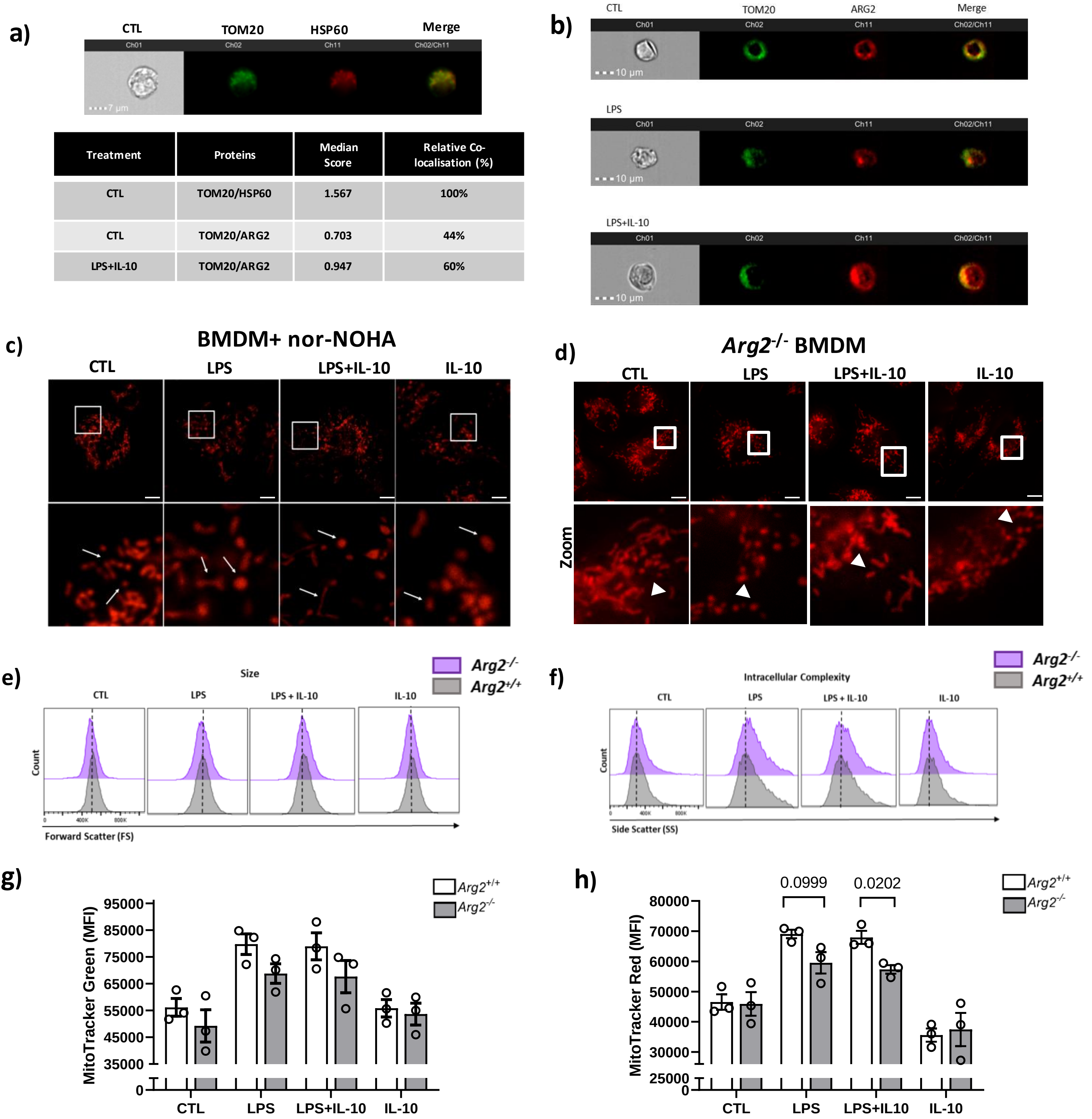

Treatment	Proteins	Median Score	Relative Co-localisation (%)
CTL	TOM20/HSP60	1.567	100%
CTL	TOM20/ARG2	0.703	44%
LPS+IL-10	TOM20/ARG2	0.947	60%

Supplementary Figure 2. Arginase-2 has limited impact on mitochondrial integrity. (a) ImageStream representative image showing co-localization levels of two mitochondrial proteins Tom20 and Hsp60 in unstimulated BMDM samples. Representative images shown from ~2000 events. Channels shown are bright field (Ch01), Tom20 (green/Ch02), Hsp60 (red/Ch11), and a merge of Tom20 and Hsp60. Original magnification, ×60. Table shows median colocalization index shown with reference index set at 100% for Tom20/Hsp60. **(b)** ImageStream representative image showing co-localization levels of Tom20 and Arg2 in unstimulated (CTL) Raw264.7 cells, or cells stimulated with LPS or LPS+IL-10. Representative image shown from ~1000 events and repeated as three independent experiments. Channels shown are bright field (Ch01), Tom20 (green/Ch02), Arg2 (red/Ch11), and a merge of Tom20 and Arg2. Original magnification, ×60. **(c)** BMDM pre-treated with 150 µM nor-NOHA, and **(d)** *Arg2*$^{-/-}$ were left unstimulated (CTL), or treated with LPS, LPS+IL-10 or IL-10 alone for 24h (full stimulation panel from Fig.2 e-f) followed by Mitotracker Red CMXRos staining for 30 min. Mitochondrial morphology was observed by confocal microscopy. Lower bottom panels show a higher magnification of the image within the white square in panels. Arrows show mitochondrial fusion and fission. **(c,d)** Data is representative of n=3 independent biological experiments. **(e-h)** *Arg2*$^{+/+}$ and *Arg2*$^{-/-}$ BMDM were untreated, or stimulated with LPS, LPS+IL-10, or IL-10 alone for 24 hours, and analysed by flow cytometry: **(e)** Representative histograms of total mitochondrial size, and **(f)** Intracellular complexity; **(g)** Mitochondrial mass by staining cells with MitoTracker Green as depicted by MFI quantification. **(h)** Membrane potential ($\Delta\Psi_m$) by staining cells with $\Delta\Psi_m$ -senstive dye MitoTracker Red as shown by MFI quantification of MitoTracker Red. **(g-h)** Data shown with error bars represents +/- SEM. Data points show three independent biological experiments. Data was analysed using two-tailed t-test with Welch's correction, and p-values for significant results are indicated on graphs.

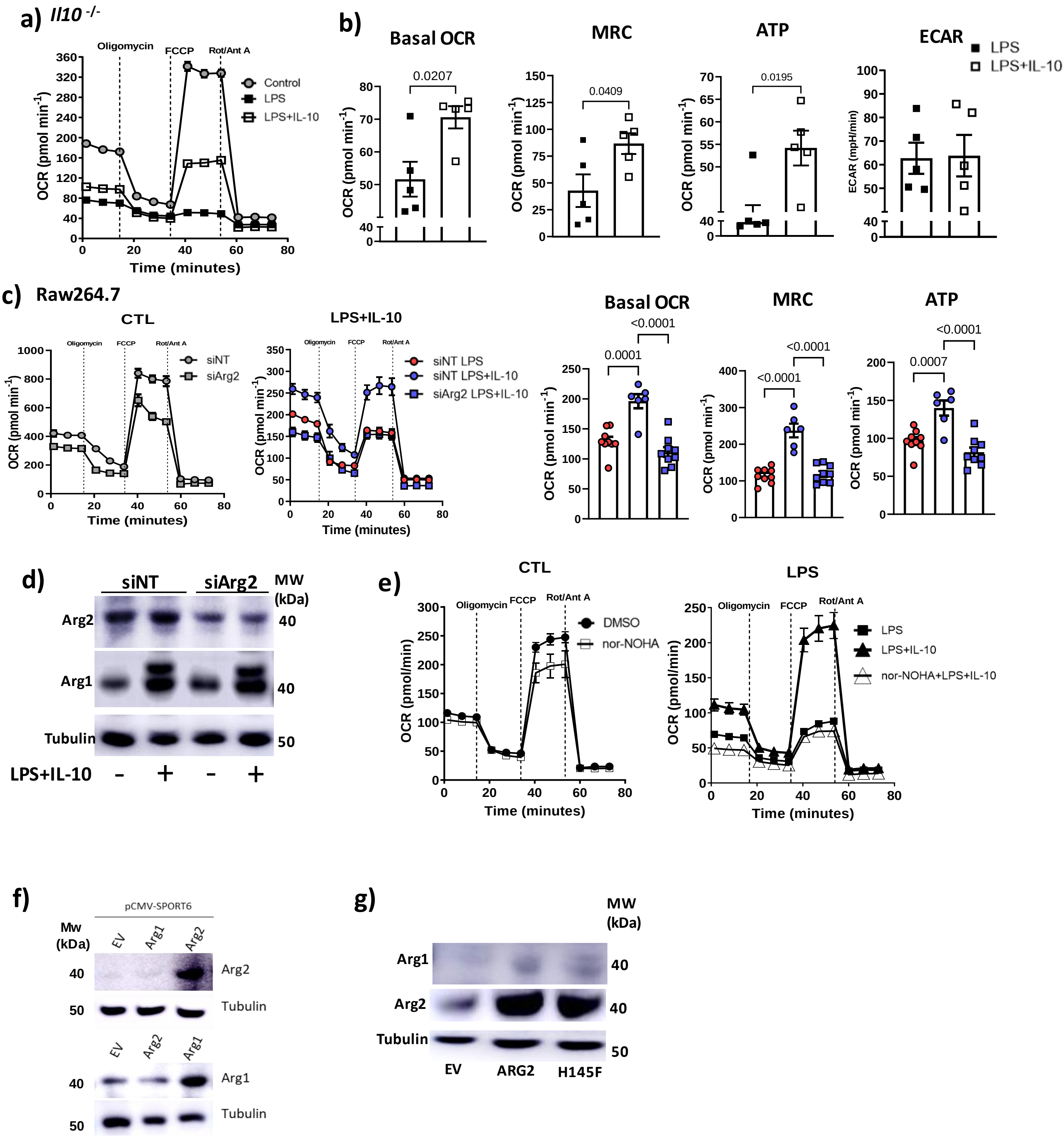
a) Il10 -/-
Oligomycin
FCCP
Rot/Ant A
Control
LPS
LPS+IL-10
OCR (pmol min-1)
Time (minutes)
b)
Basal OCR
MRC
ATP
ECAR
0.0207
0.0409
0.0195
ECAR (mpH/min)
c) Raw264.7
CTL
LPS+IL-10
siNT
siArg2
siNT LPS
siNT LPS+IL-10
siArg2 LPS+IL-10
0.0001
<0.0001
0.0007
d)
siNT
siArg2
MW (kDa)
Arg2
Arg1
Tubulin
LPS+IL-10
e)
CTL
LPS
DMSO
nor-NOHA
nor-NOHA+LPS+IL-10
OCR (pmol/min)
f)
pCMV-SPORT6
Mw (kDa)
EV
Arg1
Arg2
Tubulin
g)
EV
ARG2
H145F

Supplementary Figure 3. Arginase-2 enhances OxPhos at the mitochondria in IL-10 treated inflammatory macrophages. (a) Representative trace (n=5 biological experiments) showing real-time changes in mitochondrial OCR in *Il10*$^{-/-}$ BMDM that were left unstimulated, or stimulated with LPS or LPS+IL-10 for 24 h. **(b)** Basal OCR, ATP levels and Basal ECAR of the representative experiment from five biological replicates n=5. **(c)** Representative mitochondrial OCR trace (n=2 independent experiments) in (left) unstimulated (CTL) and (right) LPS/LPS+IL-10 stimulated Raw264.7 cells, 24 hours post stimulation. Cells were either pre-treated with DMSO or with 150 µM nor-NOHA for 1 h before addition of IL-10 and/or LPS stimulations. Scatter bar plots show quantitative changes in Raw 264.7 cells transfected with siNT or siArg2, and then stimulated with LPS or LPS+IL-10. Mean basal OCR, maximal respiratory capacity (MRC) and ATP levels are shown as pooled technical triplicates representative of three independent experiments. **(d)** iBMDM macrophages were transfected with Accell® siRNA for 72 hours against Arg2 (siArg2) and compared to non-targeting siRNA (siNT) (1 uM). Samples were then left unstimulated or stimulated with LPS+IL-10 for a further 24 hours, and looked at for protein expression of Arg2, Arg1, and tubulin as loading control (representative of n=2 independent experiments). **(e)** Representative trace of n=2 independent experiments of mitochondrial OCR in (left) unstimulated (CTL), or (right) LPS/LPS+IL-10 stimulated Raw264.7 cells, 24 hours post stimulation. Cells were either pre-treated with DMSO or with 150 µM nor-NOHA for 1 hour before addition of IL-10 and/or LPS stimulations. **(f)** Expression levels of Arg1 and Arg2, with tubulin as loading control, after transfection of pCMV-SPORT6 overexpression plasmids shown by immunoblot. Blot representative of three independent experiments. **(g)** Expression levels of Arg2 and Arg1, with tubulin as loading control, after transfection of respective pCMV-SPORT6 over-expression plasmids: Empty Vector (EV), Arg-2 and H145F are shown by immunoblot. Blot representative of two independent experiments. **(a-e)** Data is presented for all traces and scatter plots as mean ± SEM. Data was analyzed with **(b)** two-tailed paired Student's t-test, and **(c)** ordinary one-way ANOVA with Tukey's multiple comparisons post-hoc test, and significant p-values were indicated on graphs.

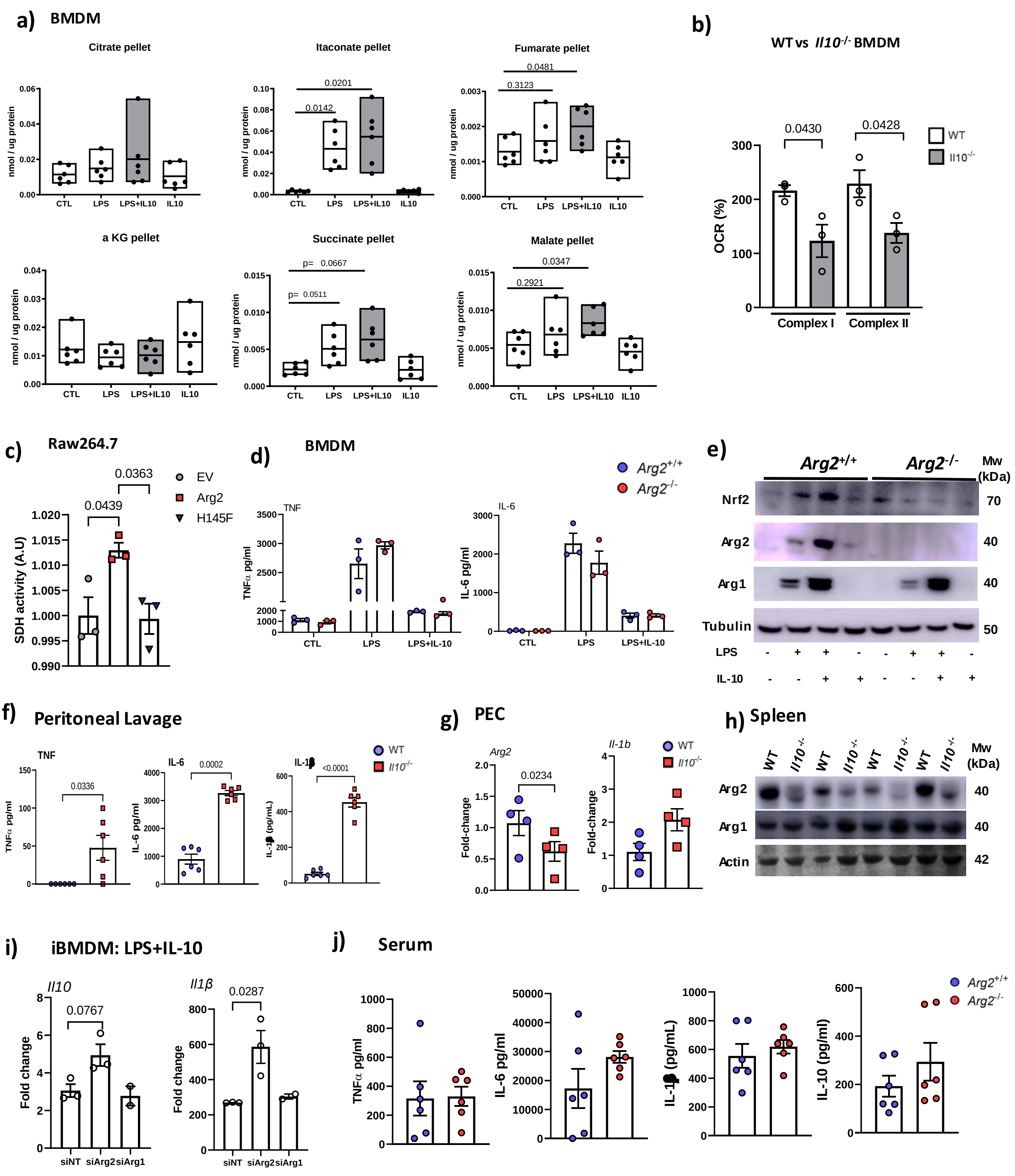

a) BMDM
Citrate pellet
Itaconate pellet
Fumarate pellet
a KG pellet
Succinate pellet
Malate pellet
nmol / ug protein
CTL
LPS
LPS+IL10
IL10
0.0201
0.0142
0.0481
0.3123
p= 0.0667
p= 0.0511
0.0347
0.2921
b)
WT vs Il10-/- BMDM
OCR (%)
0.0430
0.0428
WT
Il10-/-
Complex I
Complex II
c) Raw264.7
SDH activity (A.U)
0.0363
0.0439
EV
Arg2
H145F
d) BMDM
Arg2+/+
Arg2-/-
TNF
TNFα pg/ml
IL-6
IL-6 pg/ml
CTL
LPS
LPS+IL-10
e)
Arg2+/+
Arg2-/-
Mw (kDa)
Nrf2
Arg2
Arg1
Tubulin
LPS
IL-10
f) Peritoneal Lavage
TNF
TNFα pg/ml
0.0336
IL-6
IL-6 pg/ml
0.0002
IL-1β
<0.0001
WT
Il10-/-
g) PEC
Arg2
Fold-change
0.0234
Il-1b
WT
Il10-/-
h) Spleen
WT
Il10-/-
Mw (kDa)
Arg2
Arg1
Actin
i) iBMDM: LPS+IL-10
Il10
Fold change
0.0767
Il1β
0.0287
siNT
siArg2
siArg1
j) Serum
TNFα pg/ml
IL-6 pg/ml
IL-1β (pg/mL)
IL-10 (pg/ml)
Arg2+/+
Arg2-/-

Supplementary Figure 4. Arginase-2 is important for CII activity and IL-10 regulated IL-1β secretion in inflammatory macrophages. (a) GC/MS analysis of TCA metabolites in BMDM left untreated (CTL) or stimulated with LPS, LPS+IL-10, or IL-10 for 24 hours. (Biological n=6); **(b-d)** XFe96 Complex activity assay: Plasma membrane-permeabilized cells were provide with a cocktail of specific substrates to measure complex-II mediated respiratory activity. Representative trace showing respective boost in OCR% after succinate/rotenone+ADP addition: **(b)** Unstimulated wild type (*Il10*$^{+/+}$) vs *Il10*$^{-/-}$ BMDM Complex I and Complex II OCR% comparison (n=3 biological). **(c)** SDH activity assessed by MTT assay in pCMV-SPORT6 EV, Arg2, and H145F transfected Raw264.7 cells. Data points show 3 independent experiments. **(d-e)** Wild-type (*Arg2*$^{+/+}$) vs *Arg2*$^{-/-}$ BMDMs that were either left untreated CTL, or stimulated with LPS, LPS+IL-10 or IL-10 alone for 24 hours were looked for: **(d)** TNF and IL-6 secretion levels in supernatant (n=3), **(e)** Protein expression for Nrf2, Arg2, Arg1, and tubulin (as loading control) via immunoblotting. Representative of three independent experiments. **(f-h)** *Il10*$^{+/+}$ and *Il10*$^{-/-}$ C57Bl/6 mice were given i.p injection of LPS at 10 mg/kg for 8 hours and looked at for **(f)** Peritoneal Lavage cytokine levels (n=6), **(g)** *Arg2* and *Il-1β* gene expression in peritoneal cells (n=4). **(h)** Arg2, Arg1, and tubulin protein levels in spleen. Blot representative of six mice each from *Il10*$^{+/+}$ and *Il10*$^{-/-}$**.** **(i)** *Il-1b* and *Il-10* gene expression in immortalised BMDM (iBMDM) that were transfected with 1 uM Accell siRNA (siNT vs siArg2) for 48 hours, followed by stimulation with LPS+IL-10 for 24 hours. Data representative of n=3 independent experiments (n=2 for siArg1). **(j)** WT (*Arg2*$^{+/+}$) and *Arg2*$^{-/-}$ C57Bl/6 mice were given i.p injection of LPS at 10 mg/kg for 8 hours and looked at for serum cytokines TNF, IL-6, IL-10 and IL-1b expression levels. (n=6)**. (a-d, f-g, i-j)** Data is presented as mean ± SEM. Data was analyzed by **(a,c,i)** one-way ANOVA with Tukey's multiple comparison post-hoc test; **(b,f,g,j)** two-tailed t-test. p-values are given on the graphs where statistically significant and/or relavant.

Supplementary Figure 5

(a)

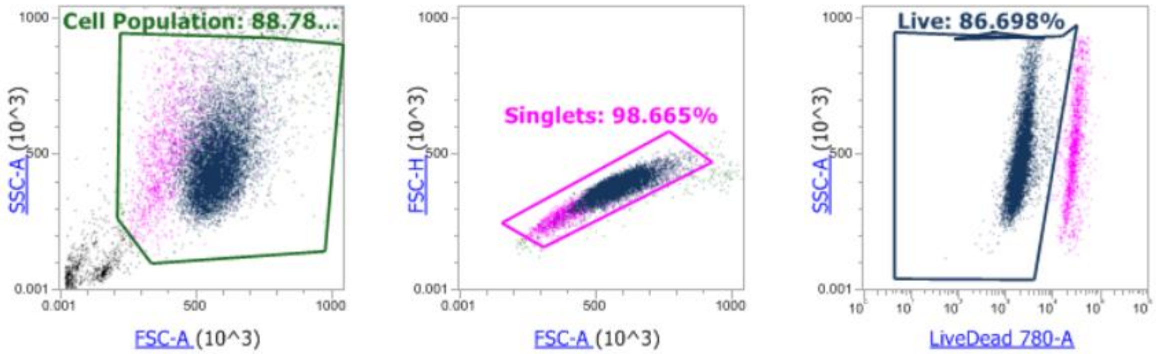

(b)

Time Recorded: **13:28:30**

Name	Gate	X Parameter	Y Parameter	Count	%Total	%Gated
All Events	All Events	N/A	N/A	13,242	100.000	100.000
Cell Population	Cell Population	FSC-A	SSC-A	11,757	88.786	88.786
Singlets	Singlets	FSC-A	FSC-H	11,600	87.600	98.665
Live	Live	RL3-A	SSC-A	10,057	75.948	86.698

Supplementary Figure 5: Gating strategy in macrophages

(a) Gating strategy for the analysis of BMDM. Cell gate population, doublet discrimination and live/dead staining. (**b**) Live events (10,000) were acquired in all experiments, Fig 4j and Supplemental Fig. 2e-f.

Supplementary Table 1. List of Antibodies, bacterial strains, and cell lines.

REAGENT or RESOURCE	SOURCE	IDENTIFIER
Antibodies		
Arginase-1 (anti-rabbit) (1:1000)	Invitrogen	Cat# PA5-85267,RRID:AB_2792410
Arginase-2 (anti-human) (1:1000)	Abcam	Cat# ab137069, RRID:AB_2848115
Arginase-2 (anti-mouse) (1:1000) and (1:100 Imagestream)	Invitrogen	Cat# MA527815 RRID:AB_2735112
Tubulin (anti-rat) (1:8000)	Abcam	Cat# ab6160, RRID:AB_305328
VDAC1 (anti-rabbit) (1:8000)	CST	Cat# 4661, RRID:AB_10557420
TOM20 (anti-rabbit) (1:200)	CST	Cat# 42406 RRID: AB_2687663
HSP60 (anti-rabbit) (1:200)	Abcam	Cat# ab5478 RRID: AB_304921
HIF-1α (anti-rabbit) (1:500)	CST	Cat# 14179, RRID:AB_2622225
Beta Actin (anti-mouse) (1:8000)	Sigma	Cat# A5441, RRID:AB_476744
NRF2 (anti-rabbit) (1:1000)	CST	Cat# 12721T RRID: AB_2715528
CD210 (IL-10 R) (anti-mouse)	BioLegend	Cat# 112707, RRID:AB_313520
Peroxidase-AffiniPure Goat Anti-Mouse IgG (H + L)	Jackson Immunoresearch	Cat#115035003 RRID:AB_10015289
Peroxidase-AffiniPure Goat Anti-Rabbit IgG (H+L)	Jackson Immunoresearch	Cat# 111-035-003,RRID:AB_2313567
Peroxidase-AffiniPure Goat Anti-Rat IgG (H+L)	Jackson Immunoresearch	Cat# 112-035-003,RRID:AB_2338128
Goat anti-Mouse IgG (H+L), Alexa Fluor Plus 488	Invitrogen	Cat# A32723
Goat anti-Rabbit IgG (H+L), Alexa Fluor Plus 488	Invitrogen	Cat# A32731
Bacterial strains		
Subcloning Efficiency™ DH5α™ Competent Cells	Invitrogen	18265017
XL10 Gold E.coli	Agilent	210518
Cell lines		
Raw 264.7	ATCC	Cat# ATCC TIB-71 RRID:CVCL_0493
L929	ATCC	Cat# ATCC CCL-1 RRID:CVCL_0462
THP-1	ATCC	Cat# TIB-202, RRID:CVCL_0006

Supplementary Table 2. List of primers

Cloning and site-directed mutagenesis	
*Arg2*_cloning_Forward	AAC GAG CTC GCT AGC CTC GAG GAA ATA CTG TAC TCT GGC AC
*Arg2*_cloning_Reverse:	CAG GTC GAC TCT AGA CTC GAG TAT GAT ATA CTA AGG TAA TAA ATG
pmir_seq_Forward:	GTG GTG TTG TGT TCG TGG AC
pmir_seq_Reverse:	CAG CCA ACT CAG CTT CCT TT
T7 promoter primer (Reverse) for pCMV-SPORT6:	TAA TAC GAC TCA CTA TAG GG
SP6 promoter (forward) for pCMV-SPORT6:	ATT TAG GTG ACA CTA TAG
H145F_Forward	CAG ATC TCT GTG TCA TCT GGG TTG ATG CTT TCG CGG ACA TTA ATA CAC
H145F_Reverse	GTG TAT TAA TGT CCG CGA AAG CAT CAA CCC AGA TGA CAC AGA GAT CTG
ASO _forward_WT_Arg2	TCA TCT GGG TTG ATG CTC AT
ASO_forward_H145F_Arg2	TCA TCT GGG TTG ATG CTT TC
RT-PCR	
Mouse_*Arg1* Forward	GTG AAG AAC CCA CGG TCT GT
Mouse_*Arg1* Reverse	CTG GTT GTC AGG GGA GTG TT
Mouse_*Arg2* Forward	GGA TCC AGA AGG TGA TGG AA
Mouse_*Arg2* Reverse	AGA GCT GAC AGC AAC CCT GT
Mouse_*Il1β* Forward	CAA CCA ACA AGT GAT ATT CTC CAT G
Mouse_*Il1β* Reverse	GAT CCA CAC TCT CCA GCT GCA
Mouse_*Il10* Forward	CAG AGA AGC ATG GCC CAG AA
Mouse_*Il10* Reverse	AGA AAT CGA TGA CAG CGC CT
Mouse_*Stat3* Forward	TCG CTC ACG TTT GAC ATG GA
Mouse_*Stat3* Reverse	CTT CTG CTC TCA GCC CCA TC
Mouse_*18s* Forward	GTA ACC CGT TGA ACC CAT T
Mouse_*18s* Reverse	CGA ATC GAA TCG GTA GTA GCG
Human_*Arg2*_Forward	TCA GTG CTG CGG ATC ATG T
Human_*Arg2*_Reverse	CAC TCC TTT TCT TTT CTG CCC TT
Human_*Arg1*_Forward	ACA AAA CAG GGC TAC TCT CAG G
Human_*Arg1*_Reverse	CGA GCA AGT CCG AAA CAA GC
Human_*18s*_Forward	GGC GCC CCC TCG ATG CTC TTA G
Human_*18s*_Reverse	GCT CGG GCC TGC TTT GAA CAC TCT

Supplementary Table 3. List of Chemical Reagents

Chemical	Catalogue	Company
1-STEP Ultra TMB ELISA Substrate	34029	ThermoFisher
Adenosine 5-diphosphate sodium salt	A2754	Sigma
2,6-Dichlorophenolindophenol (DCPIP)	D1878	Sigma
3-(4,5-dimethylthiazol-2-yl)-2,5-diphenyltetrazolium bromide (MTT formazan)	M2003	Sigma
20X LumiGLO® Reagent and 20X Peroxide	7003S	CST
Acetyl CoA	A2056	Sigma
Agarose UltraPure™	16500100	Invitrogen
Agarose gel loading dye (6X) glycerol based, Xylene Cyanol FF free	15480157	Sigma
Ampicillin sodium salt	A9518	Sigma
Atpenin A5	11898	Cayman Chemicals
BOLT® SDS Running Buffer (20X)	B0001	Invitrogen
BOLT® 4-12% Bis-Tris Plus Gels	NW04125BOX	Invitrogen
Bovine Serum Albumin (BSA)	A2153	Sigma
Bromophenol Blue	B0126	Sigma
cOmplete™, Mini, EDTA-free Protease Inhibitor Cocktail	4693159001	Roche
D-(+)-Glucose	G8769	Sigma
Decylubiquinone	D7911	Sigma
Diethylpyrocarbonate (DEPC) water	AM9920	ThermoFisher
Digitonin	BN2006	Invitrogen
Dimethyl Sulfoxide (DMSO)	D2650	Sigma
D-Mannitol	M4125	Sigma
Dithiothreitol (DTT) molecular biology grade	D0632	Sigma
DTNB	22582	ThermoFisher
Dulbeccos Modified Eagle Medium High Glucose 4500mg/L, L-glut, SB	D5796	Sigma
Dulbeccos Phosphate Buffer Saline (DPBS)	D8537	Sigma
eBioscience™ Fixable Viability Dye eFluor™ 780	65-0865-14	Invitrogen
Ethylenediaminetetraacetic acid (EDTA)	E6758	Sigma
EGTA	E3889	Sigma
Fatty acid-Free Bovine Serum Albumin	A8806	Sigma
Fetal Bovine Serum (Heat-inactivated) Batch Number #BCBX1241	F9665	Sigma
GeneRuler 100 b.p DNA ladder	SM0243	ThermoFisher
GeneRuler 1 kb Plus DNA Ladder	SM1334	ThermoFisher
Glycerol	G5516	Sigma
Glycine	G7126	Sigma
Griess' reagent for nitrite	G4410	Sigma
HEPES	H0887	Sigma
KAPA2G Fast HotStart Ready Mix	KK5601	Invitrogen
LB Broth with agar	L7025-100TAB	Sigma
Leupeptin	L2884	Sigma
L-Glutamic Acid	G1251	Sigma
L-Glutamine	G7513	Sigma
Lipofectamine® 3000 Transfection Reagent	L3000008	Invitrogen
Lipopolysaccharide (O111:B4) (Ultrapure for *in vivo* experiments)	TLRL3PELPS	Invivogen
Lipopolysaccharide (O111:B4)	ALX-581-012-L002	Enzo LifeSciences
L-Malic Acid	M7397	Sigma
Mannitol	M9647	Sigma
Magnesium Chloride	M1028	Sigma
Marvel® skimmed milk powder	B07P5DZNLN	Amazon
Methanol	34860	Sigma
mir155 inhibitor	4464084	ThermoFisher
mirVana miR-155-5p mimic	4464066	ThermoFisher
MitoSOX™	M36008	Invitrogen
MitoTracker Green	M7514	Invitrogen
MitoTracker Red CMXRos	M7512	Invitrogen

nor-NOHA (acetate)	CAY10006861	Cayman Chemicals
NP-40 Igepal	18896	Sigma
Oxaloacetate	O7753	Sigma
PageRuler Prestained Protein Ladder	26619	Invitrogen
Paraformaldehyde 4%	R37814	Invitrogen
Penicillin/Streptomycin	P4333	Sigma
Pepstatin A	P4265	Sigma
Phorbol 12-myristate 13-acetate	P8139	Sigma
PMSF	P7626	Sigma
Potassium Phosphate (dibasic)	P3786	Sigma
Potassium Phosphate (monobasic)	P8709	Sigma
PowerUp™ SYBR® Green Master Mix	A25776	Applied Biosystems
Pyruvate	S8636	Sigma
Q5 High-Fidelity DNA Polymerase - 100 Units	M0491S	NEB
Recombinant human IL-10	217-IL-005	R&D Systems
Recombinant mouse IL-10	417-ML-005	R&D Systems
Red Blood Cell Lysing Buffer Hybri-Max	R7757	Sigma
Rnase Inhibitor	N8080119	Applied Biosystems
RPMI-1640 medium	R8758	Sigma
SmaI	R0141S	NEB
Sodium Chloride	S7653	Sigm
Sodium dodecyl sulphate (SDS) 20%	05030	Sigma
Sodium hydroxide (NaOH)	221465	Sigma
Sodium orthovanadate	S6508	Sigm
Sodium phosphate dibasic heptahydrate ($Na_2HPO4 \bullet 7H_2O$)	431478	Sigma
Sodium phosphate monobasic dihydrate ($NaH_2PO_4 \bullet 2H_2O$)	71505	Sigma
STAT3 Inhibitor V, Stattic	573099	Sigma
Succinic Acid	S9512	Sigma
Sucrose	S0389	Sigma
Sulfuric Acid	339741	Sigma
SYBR™ Safe DNA Gel Stain	S33102	Invitrogen
TaqMan® Fast Advanced Master Mix	4444557	ThermoFisher
TransIT-X2 Dynamic Delivery System	MIR6003	Mirus Bio
TRI-Reagent	T9424	Sigma
Triton X-100	X100	Sigma
Trizma Base	T1503	Sigma
Trypan Blue Stain (0.4%)	T10282	Invitrogen
Trypsin-EDTA	T4174	Sigma
Tween 20	P1379	Sigma
UltraPure™ Agarose	16500100	Sigma
UltraPure™ TAE Buffer	15558042	Sigma
XF Base Medium (No Phenol Red) DMEM, pH 7.4	103575-100	Agilent
XhoI	R0146S	NEB

Supplementary Table 4. List of used commercial kits

Commercial kit	Catalogue number	Company
Accell Mouse Arg1 Set of 4, 2 nmol	(11846) EQ-043749-00-0002	Dharmacon
Accell Mouse Arg2 Set of 4, 2 nmol	(11847) EQ-040721-00-0002	Dharmacon
Accell Mouse Control siRNA Kit - Red, 5 nmol	K-005000-R1-02	Dharmacon
Arginase Assay kit	MAK112-1KT	Sigma
BCA Assay	10678484	ThermoFisher Scientific
CloneEZ® PCR Cloning Kit	L00339	Genscript
Cytometric Bead Array (CBA)	339199	BD Biosciences
Dual-Luciferase Reporter Assay System	E1910	Promega
DuoSet ELISA IL-1β	DY401-05	R&D Systems
DuoSet ELISA IL-6	DY406	R&D Systems
DuoSet ELISA IL-10	DY417-05	R&D Systems
DuoSet ELISA TNF	DY410	R&D Systems
Fumarate ChromaDazzle Activity Assay	BA0110	AssayGenie
High Capacity cDNA Reverse Transcription Kit	4368814	Applied Biosystems
Plasmid Midi Kit	12143	Qiagen
QuikChange Lightning site-directed mutagenesis	210518	Agilent
RNeasy mini kit	74106	Qiagen
Succinate Assay kit	ab204718	Abcam
TaqMan MicroRNA Reverse Transcription Kit	4366596	Applied Biosystems
XF Cell Mito Stress Test Kit	103015-100	Agilent
XFe96 Flux Pak mini kit	102601-100	Agilent
