# Supplementary material for: Mitochondrial arginase-2 is essential for IL-10 metabolic reprogramming of inflammatory macrophages
Source: Nat Commun. 2021 Mar 5;12:1460. doi: 10.1038/s41467-021-21617-2 (PMC7936006; doi:10.1038/s41467-021-21617-2)
Supplement: Supplementary file 3 — Reporting summary [file 41467_2021_21617_MOESM3_ESM.pdf]

nature research

Corresponding author(s): Claire McCoy

Last updated by author(s): Jan 20, 2021

Reporting Summary

Nature Research wishes to improve the reproducibility of the work that we publish. This form provides structure for consistency and transparency in reporting. For further information on Nature Research policies, see our Editorial Policies and the Editorial Policy Checklist.

Statistics

For all statistical analyses, confirm that the following items are present in the figure legend, table legend, main text, or Methods section.

n/a	Confirmed	
☐	☒	The exact sample size (n) for each experimental group/condition, given as a discrete number and unit of measurement
☐	☒	A statement on whether measurements were taken from distinct samples or whether the same sample was measured repeatedly
☐	☒	The statistical test(s) used AND whether they are one- or two-sided<br>*Only common tests should be described solely by name; describe more complex techniques in the Methods section.*
☐	☒	A description of all covariates tested
☐	☒	A description of any assumptions or corrections, such as tests of normality and adjustment for multiple comparisons
☐	☒	A full description of the statistical parameters including central tendency (e.g. means) or other basic estimates (e.g. regression coefficient) AND variation (e.g. standard deviation) or associated estimates of uncertainty (e.g. confidence intervals)
☐	☒	For null hypothesis testing, the test statistic (e.g. F, t, r) with confidence intervals, effect sizes, degrees of freedom and P value noted<br>*Give P values as exact values whenever suitable.*
☒	☐	For Bayesian analysis, information on the choice of priors and Markov chain Monte Carlo settings
☒	☐	For hierarchical and complex designs, identification of the appropriate level for tests and full reporting of outcomes
☒	☐	Estimates of effect sizes (e.g. Cohen's d, Pearson's r), indicating how they were calculated

Our web collection on statistics for biologists contains articles on many of the points above.

Software and code

Policy information about availability of computer code

Data collection	Affymetrix microarray was performed by Almac diagnostics.<br>Applied Biosystems 7500 or 7900 HT was used to collect RT-PCR data<br>XFe96 analyzer (Agilent) was used to perform metabolic assays.<br>Amersham Imager 600 was used to collect western blot images.<br>Imagestream MK-II cytometer (Amnis) was used to collect imagestream data.<br>Leica SP8 scanning confocal microscopy with a 63x immersion objective was used to get confocal images.<br>Attune NxT Flow Cytometer was used to obtain FACs data.<br>VICTOR3 Multilabel Plate Reader was used for ELISAs.
Data analysis	GraphPad PRISM 8 was used to perform statistical analysis and plot results. Metabolic flux assays were analysed using the Wave (Agilent) Software. ImageStudiolite was used to perform western blot quantifications. IDEAS (Image Data Exploration and Analysis Software) was used to analyse imagestream flow data. 7500 SDS v1.4.1 was used to analyze RT-PCR data. FlowJo v10 software was used for FACs measurements. Mass Hunter (v B.07.01/Build 7.1.524.0) was used for GC/MS metabolomic analysis. ImageJ was used to quantify confocal data. Microarray data was analyzed using affy v. 1.58.0, R v. 3.5.1 and limma package V3.36.5. Gene set enrichment analysis was performed using the romer function using the CS Gene Ontology (GO) gene sets from Molecular Signatures Database v5.2, (references in manuscript).

For manuscripts utilizing custom algorithms or software that are central to the research but not yet described in published literature, software must be made available to editors and reviewers. We strongly encourage code deposition in a community repository (e.g. GitHub). See the Nature Research guidelines for submitting code & software for further information.

Data

Policy information about availability of data

All manuscripts must include a data availability statement. This statement should provide the following information, where applicable:

- Accession codes, unique identifiers, or web links for publicly available datasets
- A list of figures that have associated raw data
- A description of any restrictions on data availability

Data for this manuscript is available within the paper and supplementary files except for Figure 1 and Supplemental Figure 1 where the Affymetrix data has been submitted to GEO under accession number GSE151835 and is available on. https://www.ncbi.nlm.nih.gov/geo/query/acc.cgi?acc=GSE151835 A source data file is also provided with this manuscript as part of the Supplementary information.

Field-specific reporting

Please select the one below that is the best fit for your research. If you are not sure, read the appropriate sections before making your selection.

[x] Life sciences [ ] Behavioural & social sciences [ ] Ecological, evolutionary & environmental sciences

For a reference copy of the document with all sections, see nature.com/documents/nr-reporting-summary-flat.pdf

Life sciences study design

All studies must disclose on these points even when the disclosure is negative.

Sample size	No statistical method was used to predetermine sample sizes for in vitro experiments. For cell biology and metabolomics experiments, we chose sample sizes based on our previous experience (Dowling et al., J Immunol 2019; Assmann et al., Nat immunol 2017; McCoy et al., J Biol Chem 2010). For ex vivo experiments and sample analysis we determined sample size as described (Lazic et al. PLoS Biology 2018) Briefly, using a significance level (alpha) in unpaired t-test with a Power value less than 0.05 (two-tailed) a statistical power of 95% can be detected with group sizes of n=5-10. Therefore, we used n=4-6 in our ex vivo experimental studies as reported previously by ourselves (Dowling et al., J Immunol 2019) and others Zasłona et al., Nat Comm 2020).
Data exclusions	Data was not excluded
Replication	For all experimental results were replicated at least two-three independent times and biological replicates of at least 3 were used for BMDM experiments. For metabolic assay studies each biological replicate had at least 3 technical replicates For Raw264.7 and iBMDM studies, more than 6 technical replicates were used per experiment for metabolic assays. Each figure contains details of experimental replicates in the figure legend that were reproduced successfully.
Randomization	Mice were randomly assigned to each experimental group. Female littermate mice of C57Bl/6J background were used at 8-12 weeks of age for BMDM generation and in vivo LPS challenge. For GC-MS data collection, sample runs were randomized in order to limit analytical variance due to retention time drifts.
Blinding	The investigators were blinded to allocation and outcome assessment of experiments involving animal studies except for Western blotting of ex vivo samples when loading had to be allocated on the gel. For experiments other than animal studies, blinding was not possible because data were largely analyzed by individual scientists who both collected and processed the samples.

Reporting for specific materials, systems and methods

We require information from authors about some types of materials, experimental systems and methods used in many studies. Here, indicate whether each material, system or method listed is relevant to your study. If you are not sure if a list item applies to your research, read the appropriate section before selecting a response.

Materials & experimental systems

n/a	Involved in the study	
[ ]	[x]	Antibodies
[ ]	[x]	Eukaryotic cell lines
[x]	[ ]	Palaeontology and archaeology
[ ]	[x]	Animals and other organisms
[x]	[ ]	Human research participants
[x]	[ ]	Clinical data
[x]	[ ]	Dual use research of concern

Methods

n/a	Involved in the study	
[x]	[ ]	ChIP-seq
[ ]	[x]	Flow cytometry
[x]	[ ]	MRI-based neuroimaging

Antibodies

Antibodies used	Invitrogen Arginase-1 (RRID:AB_2792410), Invitrogen Arginase-2 (RRID:AB_2735112), Abcam Tubulin (RRID:AB_305328), CST VDAC1 (RRID:AB_10557420), CST TOM20 (RRID:AB_2687663), Abcam HSP60 (RRID:AB_304921), CST NRF2 (RRID:AB_2715528). Jackson Immunoresearch secondary antibodies used: Peroxidase-AffiniPure Goat Anti Mouse IgG (H+L) (RRID: AB_10015289), Peroxidase-AffiniPure Goat Anti Rabbit IgG (H+L) (RRID: AB_2313567), Peroxidase-AffiniPure Goat Anti Rat IgG (H+L) (RRID: AB_2338128), Invitrogen Goat Anti Mouse IgG (H+L) Highly Cross Adsorbed Secondary, Alexa Fluor Plus 488 (Cat# A32723)and 647 (Cat #A32728). Invitrogen Goat Anti Rabbit IgG (H+L) Highly Cross Adsorbed Secondary, Alexa Fluor Plus 488 (Cat# A32731) and 647 (Cat# A32733). Abcam Rabbit anti Human Arginase-2 (Cat# Ab137069}, Cell Signaling Rabbit Anti Mouse HIF-lalpha (Cat# 141795), Sigma Mouse Anti beta-actin (Cat# A5441). Further Details of the catalogue numbers for primary antibodies and concentration used for each antibody can be found in supplementary resources table.
Validation	All antibodies employed in our manuscript were previously reported and routinely used for the application herein used. All vendors used (Invitrogen, Abcam, Sigma, Cell Signalling Technology (CST), Jackson Immunoresearch) report taking quality control measures to ensure that all antibodies sold are valid and reproducible. Information and citations for each antibody used herein could be found in the corresponding data sheets provided by the commercial supplier and visiting the company's on-line repositories. Catalogue numbers for all antibodies used herein can be found in the Source data file (last tab).

Eukaryotic cell lines

Policy information about cell lines

Cell line source(s)	Raw 264.7 (ATCC TIB-71; RRID: CVCL_0493), NCTC clone 929 [L-cell, L929] (ATCC CCL-1; RRID: CVCL_0462), THP-1 (ATCC TIB-202; RRID: CVCL_0006).
Authentication	Cell lines were authenticated by manufacturer (ATCC) and confirmed by microscopy looking at morphology. L929 cells were confirmed to produce MCSF as determined by ELISA.
Mycoplasma contamination	Cell lines in the School Pharmacy and Bimolecular Sciences were Mycoplasma tested monthly and found to be negative.
Commonly misidentified lines (See ICLAC register)	No commonly misidentified cell lines were used in this study.

Animals and other organisms

Policy information about studies involving animals; ARRIVE guidelines recommended for reporting animal research

Laboratory animals	All mice were bred and maintained in on-site barrier controlled facilities under a 12 h light–dark cycle at room temperature (~22°C), maintained at 30–70% relative humidity, and provided with food and water ad libitum.<br>C57Bl/6J (Jax labs stock number: 664), miR-155 deficient, (Jax Labs Stock No: 007745) and Arg2 deficient mice (Jax labs stock number: 020286) were all purchased from Jackson labs. IL-10 deficient mice were kindly provided by Prof. Ed Lavelle of Trinity College Dublin, and were also originally purchased from Jackson labs and bred in the Bio resources unit of Trinity Biomedical Sciences Institute Female mice were used at 8-12 weeks of age for experiments.
Wild animals	Not used
Field-collected samples	Not used
Ethics oversight	Procedures were approved by the Ethics Committee of the Royal College of Surgeons in Ireland (REC-842), under license from the Ireland Health Products Regulatory Authority (AE19127/001). IL-10 deficient mice bred in Trinity Biomedical Sciences Institute had Ethics Approval number:091210 and License number: AE19136/P079. Arg-2 deficient mice were housed in Biological Research Facility (BRF) at RCSI under license: A19127 /P045. All procedures conformed to the Directive 2010/63 EU of the European Parliament.

Note that full information on the approval of the study protocol must also be provided in the manuscript.

Flow Cytometry

Plots

Confirm that:

- [x] The axis labels state the marker and fluorochrome used (e.g. CD4-FITC).
- [x] The axis scales are clearly visible. Include numbers along axes only for bottom left plot of group (a 'group' is an analysis of identical markers).
- [ ] All plots are contour plots with outliers or pseudocolor plots.
- [x] A numerical value for number of cells or percentage (with statistics) is provided.

Methodology

Sample preparation	BMDM were seeded in low adherence culture plates and stimulated as described in methods section. Cells were stained with Mitotracker green, Mitotracker Red, or MitoSOX (all Invitrogen) according to manufacturers instructions . Cytokines levels in mouse serum were measured using the cytometric bead array, mouse inflammation kit (BD BioSciences).
Instrument	Attune NxT Flow Cytometer
Software	FlowJo v10
Cell population abundance	Following differentiation with 20% (v/v) L929 conditioned media the adherent cell population was confirmed positive for BMDM marker CD11b.
Gating strategy	In each experiment singlet cells were identified from the total cell population and dead cells were excluded by Live/Dead stain, eBioscience'M Fixable Viability Dye eFluor'" 780.

☒ Tick this box to confirm that a figure exemplifying the gating strategy is provided in the Supplementary Information.
